# Cholera Cured by the Actea Racemosa

**Published:** 1833

**Authors:** 


Art. IV. *Chorea cured by the Actea Racenosa.* In the same periodical, the same physician has borne testimony to these effects of the Actea in *Chorea St. Viti.* A boy of 17 had this disease in one side only. His health otherwise seemed perfectly good. Without the least preparation, by the lancet, cathartic, or other means, he was put on the use of a tea-spoonful of the powdered root, three times a day. He was getting worse at the time; but was visibly better in two days, and, in five, entirely cured. Dr. Young has treated others in the same way, with the same result.

